# The IκB kinase inhibitor ACHP strongly attenuates TGFβ1‐induced myofibroblast formation and collagen synthesis

**DOI:** 10.1111/jcmm.12661

**Published:** 2015-09-04

**Authors:** Masum M. Mia, Ruud A. Bank

**Affiliations:** ^1^ Department of Pathology and Medical BiologyDivision of Medical BiologyUniversity of GroningenUniversity Medical Center GroningenGroningenThe Netherlands

**Keywords:** fibrosis, collagen, myofibroblasts, fibronectin, lysyl hydroxylase

## Abstract

Excessive accumulation of a collagen‐rich extracellular matrix (ECM) by myofibroblasts is a characteristic feature of fibrosis, a pathological state leading to serious organ dysfunction. Transforming growth factor beta1 (TGFβ1) is a strong inducer of myofibroblast formation and subsequent collagen production. Currently, there are no remedies for the treatment of fibrosis. Activation of the nuclear factor kappa B (NF‐κB) pathway by phosphorylating IκB with the enzyme IκB kinase (IKK) plays a major role in the induction of fibrosis. ACHP {2‐Amino‐6‐[2‐(cyclopropylmethoxy)‐6‐hydroxyphenyl]‐4‐(4‐piperidinyl)‐3 pyridinecarbonitrile}, a selective inhibitor of IKK, prohibits the activation of the NF‐κB pathway. It is not known whether ACHP has potential anti‐fibrotic properties. Using adult human dermal and lung fibroblasts we have investigated whether ACHP has the ability to inhibit the TGFβ1‐induced transition of fibroblasts into myofibroblasts and its excessive synthesis of ECM. The presence of ACHP strongly suppressed the induction of the myofibroblast markers alpha‐smooth muscle actin (αSMA) and SM22α, as well as the deposition of the ECM components collagen type I and fibronectin. Furthermore, post‐treatment with ACHP partly reversed the expression of αSMA and collagen type I production. Finally, ACHP suppressed the expression of the three collagen‐modifying enzymes lysyl hydroxylase (*PLOD1*,*PLOD2* and *PLOD3*) in dermal fibroblasts, but did not do so in lung fibroblasts. We conclude that the IKK inhibitor ACHP has potent antifibrotic properties, and that the NF‐κB pathway plays an important role in myofibroblast biology.

## Introduction

Fibrosis is a common outcome of an impaired tissue repair process. The hallmark of fibrosis is the production and excessive accumulation of a collagen‐rich extracellular matrix (ECM). The ECM is deposited by myofibroblasts under the influence of pro‐fibrotic cytokines, such as transforming growth factor beta 1 (TGFβ1) 1, 2. TGFβ1 is one of the most potent pro‐fibrotic cytokines known to be involved in the activation of fibroblasts into myofibroblasts, a key process associated with fibrosis that seems to be dependent, among others, on the activation of the nuclear factor kappa B (NF‐κB) pathway 3, 4, 5, 6, especially NF‐κB subunit p65 (= RelA).

The RelA signalling pathway is associated with the pathogenesis of fibrosis including organs such as kidney, liver and lung 5, 6, 7, 8, 9, 10, 11, 12, 13. In unstimulated cells, the cytoplasmic NF‐κB is bound to the inhibitory protein IκB; the complex prevents NF‐κB activation (*i.e*. the complex remains in the cytoplasm). Extracellular stimuli activate the enzyme IκB kinase (IKK) that phosphorylates the IκB subunit of the NF‐κB/IκB complex. The phosphorylated IκB is degraded and NF‐κB is translocated to the nucleus, where it is able to bind to its target sequences and subsequently activate gene transcription 13, 14, 15, 16.

Activation of IKK has been reported to induce liver fibrosis in mice 12. Selective deletion of IKKβ (a catalytic subunit of the IKK complex) in mouse airway epithelium blocked the nuclear translocation of RelA and showed less peribronchial fibrosis 17. Overexpression of integrin‐linked kinase in rat cardiac fibroblasts induces a pro‐fibrotic response by increasing the production of collagen type I and connective tissue growth factor *via* RelA; transfection of cardiac fibroblasts with mutant IκBα (a member of IκB) inactivated RelA, thereby decreasing fibrosis 18. A direct approach using RelA antisense oligonucleotides reduced the formation of the myofibroblast marker alpha‐smooth muscle actin (αSMA) in bleomycin‐induced mouse lung fibrosis and in cultured cells, showing the deleterious role of NF‐κB in the development and progression of organ fibrosis 9, 19. These examples show that inhibition of the IKK/NF‐κB pathway might be an attractive therapeutic tool to attenuate fibrosis.

Several NF‐κB pathway inhibitors have been investigated in animal models to slow down the fibrotic reaction. IMD‐0354 (an IKKβ inhibitor) prevented the activation of RelA and collagen content in bleomycin‐induced lung fibrosis in mice 20. Administration of Suramin, a polysulfonated naphthylurea, inhibited the TGFβ1/Smad3 pathway and the phosphorylation of IκBα in fibrotic peritoneum and thereby reduced peritoneal fibrosis in rat 21. Salvianolic acid B, derived from *Salvia miltiorrhiza* (a Chinese herbal medicine), has been reported to reduce carbon tetrachloride‐induced liver fibrosis in rats which correlated with an increased level of RelA and IκBα protein in the cytoplasm but not in the nucleus 22. Similarly, the expression of αSMA was decreased through the inhibition of IKK with a boswellic acid‐containing extract treatment in a mouse model of *Schistosomiasis* liver granuloma and fibrosis 23. Pressure overload‐induced cardiac fibrosis has been treated with Sophocarpine, a tetracyclic quinolizidine alkaloid, resulting in a reduction of collagen deposition by inhibiting IκBα phosphorylation 24. Unfortunately, in all these studies, only limited protective effects of these agents have been described. Therefore, another therapeutic agent that interferes with the NF‐κB system is wanted to reduce or inhibit fibrosis progression.

The low molecular weight compound 2‐Amino‐6‐[2‐(cyclopropylmethoxy)‐6‐hydroxyphenyl]‐4‐(4‐piperidinyl)‐3 pyridinecarbonitrile (ACHP) is a selective inhibitor of IKK (both for the IKKα and the IKKβ subunit) 25, 26, 27. So far, no investigations have been performed to explore whether ACHP is able to interfere with fibrotic processes, such as blocking the TGFβ1‐induced transition of fibroblasts into myofibroblasts. In this study, we examined whether ACHP can directly inhibit myofibroblast formation and ECM synthesis. To decipher this, adult human dermal and lung fibroblasts (HDFa and HLFa) were stimulated with TGFβ1 in the presence or absence of ACHP and investigated the formation of myofibroblasts and the deposition of ECM‐molecules. In addition, we explored whether myofibroblasts that are formed by TGFβ1 can be reversed into fibroblasts with an ACHP post‐treatment. We found that ACHP strongly attenuates TGFβ1‐induced formation of myofibroblasts as well as collagen type I and fibronectin protein synthesis.

## Materials and methods

### Materials

Eagle's minimal essential medium (EMEM) and l‐glutamine were obtained from Lonza Group (Basel, Switzerland), penicillin/streptomycin was obtained from Gibco Life Technologies (Paisly, UK), foetal bovine serum (FBS) was obtained from Thermo Scientific (Waltham, MA, USA), bovine serum albumin (BSA) was obtained from Sanquin (Sanquin, Netherlands) and culture plates and chamber slides were obtained from Corning (Corning, NY, USA). ACHP (#4547) was purchased from Tocris (Bristol, UK), recombinant human TGFβ1 (#100‐21) from Peprotech (London, UK), and l‐ascorbic acid 2‐phosphate magnesium salt (#A‐8960) from Sigma‐Aldrich (St. Louis, MO, USA). FARB buffer and the RNA extraction kit were purchased from Favorgen Biotech (Ping‐Tung, Taiwan), the cDNA synthesis kit was from Fermentas (Vilnius, Lithuania), methanol and acetone was from Merck (Darmstadt, Germany), SYBR Green Master Mix was from Roche (Pleasanton, CA, USA), streptavidin‐CY3 was from Invitrogen (Carlsbad, CA, USA) and Citifluor was from Agar Scientific (Stansted, UK).

### Cell culture

Human adult dermal fibroblasts [Caucasian, 20 years, CCD‐1093Sk (ATCC^®^ CRL‐2115^™^), ATCC, Manassas, VA, USA] and Human adult lung fibroblasts [Caucasian, 27 years, CCD‐19Lu (ATCC^®^ CCL‐210^™^), ATCC] were cultured in basal medium (= EMEM containing 1% l‐glutamine and 1% penicillin/streptomycin) supplemented with 10% FBS. Passage 5 to 8 of HDFa and HLFa were seeded with a density of 15,000 cells/cm^2^ in a Costar 12‐well plate for quantitative real‐time polymerase chain reaction (qRT‐PCR) or in a 48‐well plate for immunofluorescence staining. After 72 hrs, fibroblasts were washed with PBS and starved overnight with basal medium containing 0.5% FBS. Subsequently, fibroblasts were treated with/without ACHP (50 μM), recombinant human TGFβ1 (10 ng/ml), or a combination of both, for a period of 24 hrs (for qRT‐PCR) and 48 hrs (for immunofluorescence staining) in basal medium supplemented with 0.17 mM l‐ascorbic acid 2‐phosphate magnesium salt and 0.5% FBS. In another experiment, fibroblasts were stimulated with TGFβ1 (10 ng/ml) for 48 hrs followed by a post‐treatment with/without ACHP (50 μM) for 24 hrs in basal medium supplemented with 0.17 mM l‐ascorbic acid 2‐phosphate magnesium salt and 0.5% FBS. Subsequently, whole‐cell lysates (as obtained with FARB‐buffer) were used for qRT‐PCR. For immunofluorescence analysis, fibroblasts were washed with PBS and fixed with methanol/acetone solution (1:1 ratio) for 5 min. The ACHP compound was dissolved in sterile water at a concentration of 12.5 mM. All cell culture protocols were performed at 37°C in a humidified 20% O_2_/5% CO_2_ environment.

### RNA isolation, cDNA synthesis and qRT‐PCR

Total RNA was isolated using the Favorgen RNA extraction kit and reverse transcribed with the First Strand cDNA synthesis kit. Gene expression analysis was performed by means of qRT‐PCR in a 10 μl reaction mixture containing 10 ng cDNA, SYBR Green Master Mix, 6 μM forward primer and 6 μM reverse primer (for primer sequences see Table 1). qRT‐PCR was conducted in triplicate for each condition in a 384‐well plate at 95°C for 15 sec. and 60°C for 1 min. for 40 cycles using the ViiA 7 Real‐Time PCR System (Applied Biosystems, Waltham, MA, USA). All mRNA data were normalized against the reference gene tyrosine 3‐monooxygenase/tryptophan 5‐monooxygenase activation protein, zeta isoform (*YWHAZ*).

**Table 1:** List of primer sequences used for qRT‐PCR

Gene	Forward sequence	Reverse sequence
*ACTA2*	CTGTTCCAGCCATCCTTCAT	TCATGATGCTGTTGTAGGTGGT
*TAGLN*	GGCCAAGGCTCTACTGTCTG	CCCTTGTTGGCCATGTCT
*COL1A1*	GCCTCAAGGTATTGCTGGAC	ACCTTGTTTGCCAGGTTCAC
*FN1*	CTGGCCGAAAATACATTGTAAA	CCACAGTCGGGTCAGGAG
*PLOD1*	GAAGCTCTACCCCGGCTACT	CTTGTAGCGGACGACAAAGG
*PLOD2*	ATGGAAATGGACCCACCAA	TGCAGCCATTATCCTGTGTC
*PLOD3*	GCTCTGCGGAGTTCTTCAAC	TAACCACCGGACCTTCTGTC
*YWHAZ*	GATCCCCAATGCTTCACAAG	TGCTTGTTGTGACTGATCGAC

### Immunofluorescence staining: αSMA, SM22α, collagen type I, fibronectin, Ki‐67

After methanol/acetone fixation, fibroblasts were washed with PBS solution and incubated with primary antibodies (αSMA: mouse monoclonal IgG2a, #M0851; Dako, Denmark; SM22α: polyclonal rabbit IgG, ab14106; Abcam, Glostrup, UK; collagen type I: mouse monoclonal IgG, ab90395; Abcam, Milton; fibronectin: rabbit polyclonal IgG, ab6584; Abcam; Ki‐67: Rabbit monoclonal IgG, ab16667; Abcam) diluted in PBS containing 2% BSA for 1 hr at RT (1:100, 1:200, 1:300 and 1:400 respectively). After washing with PBS, cells were incubated for 30 min. at RT with biotinylated secondary antibodies (αSMA: goat‐antimouse IgG2a, 1080‐08; SouthernBiotech, Birmingham, AL, USA; SM22α, Ki‐67 and fibronectin: goat‐anti‐rabbit IgG, E0432; Dako; collagen type I: goat‐antimouse IgG1, 1071‐08; SouthernBiotech) diluted in PBS (1:100) containing 2% BSA for 30 min. at RT. The cells were washed again and incubated with streptavidine‐CY3 (1:100) in PBS containing 1% BSA and (diamidino‐2‐phenylindole) DAPI (1:10,000) for 30 min. After washing with PBS, cell culture wells were mounted with Citifluor and the staining pattern was visualized with fluorescence imaging microscopy (TissueFAXS; TissueGnostics GmbH, Wien, Austria). TissueFAXS data were analysed with the TissueQuest software as described previously 28.

### Immunofluorescence staining for RelA and Smad2/3

Human dermal and lung fibroblasts were treated for 15, 30 and 45 min. with (*i*) ACHP, (*ii*) TGFβ1, or (*iii*) both. After treatment, cells were washed with PBS and fixed either with methanol/acetone (1:1) (Smad2/3) for 5 min. or with 0.5% para‐formaldehyde (RelA) for 15 min. Subsequently, cells were washed with PBS and incubated either with polyclonal goat‐anti‐human to Smad2/3 (AF3797; R&D, Abingdon, UK) diluted in a concentration of 15 μg/ml, or with polyclonal rabbit anti‐human to RelA (ab16502; Abcam) at 1:50 dilution in PBS containing 2% BSA for 3 hrs at 4°C. After washing with PBS, cells were incubated with biotinylated secondary antibody rabbit anti‐goat to detect Smad2/3 (6160‐08; SouthernBiotech) and goat‐anti‐rabbit to detect RelA (E0432; Dako) diluted in PBS (1:100) containing 2% BSA for 30 min. at RT. The cells were washed again and incubated for 30 min. with streptavidin‐CY3 (1:100) in PBS containing 1% BSA and DAPI (1:10,000). After washing with PBS, stained wells were mounted with Citifluor and the translocation of RelA and Smad2/3 was visualized by using confocal laser scanning microscopy (Leica TCS SP8; Leica Microsystems GmBH, Wetzlar, Germany).

### Statistics

All mRNA and immunofluorescence data are presented as mean ± SEM for at least three independent experiments. Results were analysed with either one‐way anova followed by Tukey's post‐test or two tailed unpaired *t*‐test analysis using Graph‐Pad Prism Version 5 (GraphPad Software, La Jolla, CA, USA). *P* < 0.05 was considered to be statistically significant. The signs * and # represents a statistically significant difference compared to the untreated, and TGFβ1 treatment respectively.

## Results

### ACHP inhibits nuclear translocation of RelA

**Figure 1:**
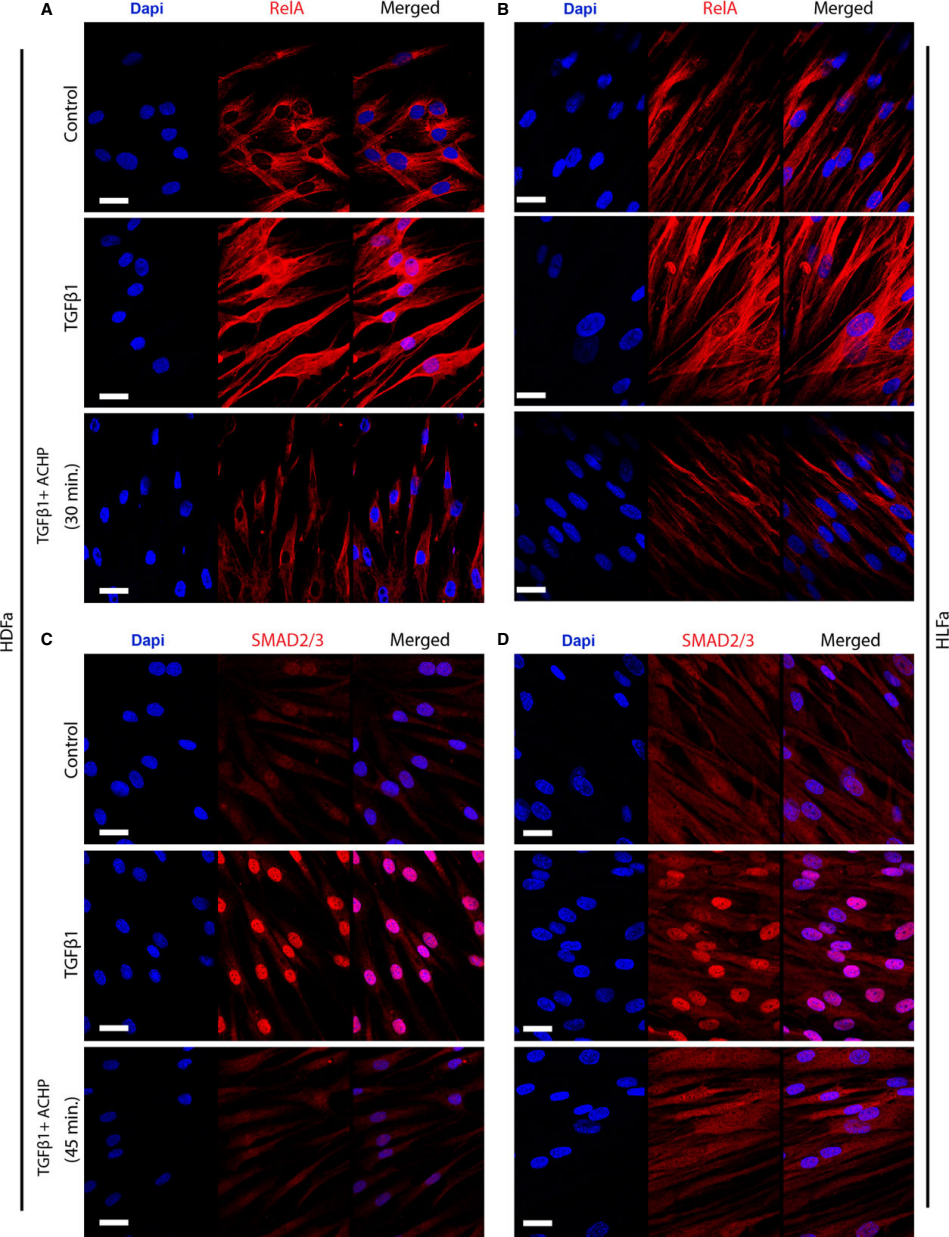
Effects of ACHP on the nuclear translocation of RelA and Smad2/3. (**A** and **B**) human dermal and lung fibroblasts (HDFa and HLFa) were cultured for 30 min. in the presence of ACHP alone, transforming growth factor beta1 (TGFβ1) alone or TGFβ1 in combination with ACHP (co‐treatment). Representative immunofluorescence stainings for the cytoplasmic and nuclear localization of RelA with confocal laser scanning microscopy. (**C** and **D**) HDFa and HLFa were cultured for 45 min. in the presence of ACHP alone, TGFβ1 alone or TGFβ1 in combination with ACHP (co‐treatment). Representative immunofluorescence stainings for the cytoplasmic and nuclear localization of Smad2/3 with confocal laser scanning microscopy. The scale bar represent 25 μm.

First, we examined whether ACHP indeed blocked the enzyme IKK. If so, RelA in HDFa and HLFa would mainly stay in the cytoplasm, instead of being translocated to the nucleus. Stimulation of HDFa and HLFa with TGFβ1 induces the nuclear translocation of RelA first at 15 min. of treatment (data not shown), and reached the highest nuclear expression after 30 min. of treatment (Fig. 1A and B). Treatment of fibroblasts with ACHP in presence of TGFβ1 efficiently inhibited the nuclear translocation of RelA in a time‐dependent manner. The inhibitory effects were seen as early as 15 min. after treatment (data not shown) and the effect was most prominent at 30 min. (Fig. 1A and B).

### Effects of ACHP on the activation of Smad2/3

Since RelA is involved in the myofibroblast differentiation process as induced by TGFβ 5, we then wondered whether a down‐stream target of TGFβ1‐signalling, namely Smad2/3, is affected by the lack of nuclear translocation of RelA. We thus evaluated the effects of ACHP on nuclear translocation of Smad2/3 in HDFa and HLFa. Confocal microscopy imaging shows that TGFβ1‐treatment activated the nuclear translocation of Smad2/3 in both types of fibroblasts already at 15 min. of incubation (data not shown) and reached the highest level at 45 min. (Fig. 1C and D). We therefore selected 45 min. to observe the effects of ACHP on Smad2/3 translocation. In a TGFβ1‐rich pro‐fibrotic environment, ACHP resulted in a blockage of the nuclear translocation of Smad2/3 in both HDFa and HLFa (Fig. 1C and D).

### Effect of TGFβ1 and ACHP on αSMA synthesis

**Figure 2:**
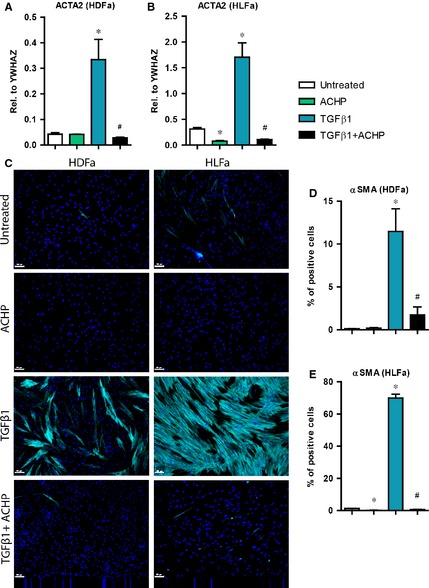
Effects of ACHP on transforming growth factor beta1 (TGFβ1)‐induced alpha‐smooth muscle actin (αSMA) synthesis. (**A** and **B**) human dermal and lung fibroblasts (HDFa and HLFa) were cultured for 24 hrs in the presence of ACHP alone, TGFβ1 alone or TGFβ1 in combination with ACHP (co‐treatment). mRNA levels of *ACTA2* relative to the reference gene *YWHAZ*. (**C**–**E**) HDFa and HLFa were cultured for 48 hrs in the presence of ACHP alone, TGFβ1 alone or TGFβ1 in combination with ACHP (co‐treatment). Representative immunofluorescence stainings (left panel) and quantification of the % of cells (right panel) positive for αSMA. The scale bars represent 100 μm. The sign * represents statistically significance towards untreated control, and the sign # represents statistically significance of cells co‐stimulated with TGFβ1 and ACHP compared to TGFβ1‐treated cells.

**Figure 3:**
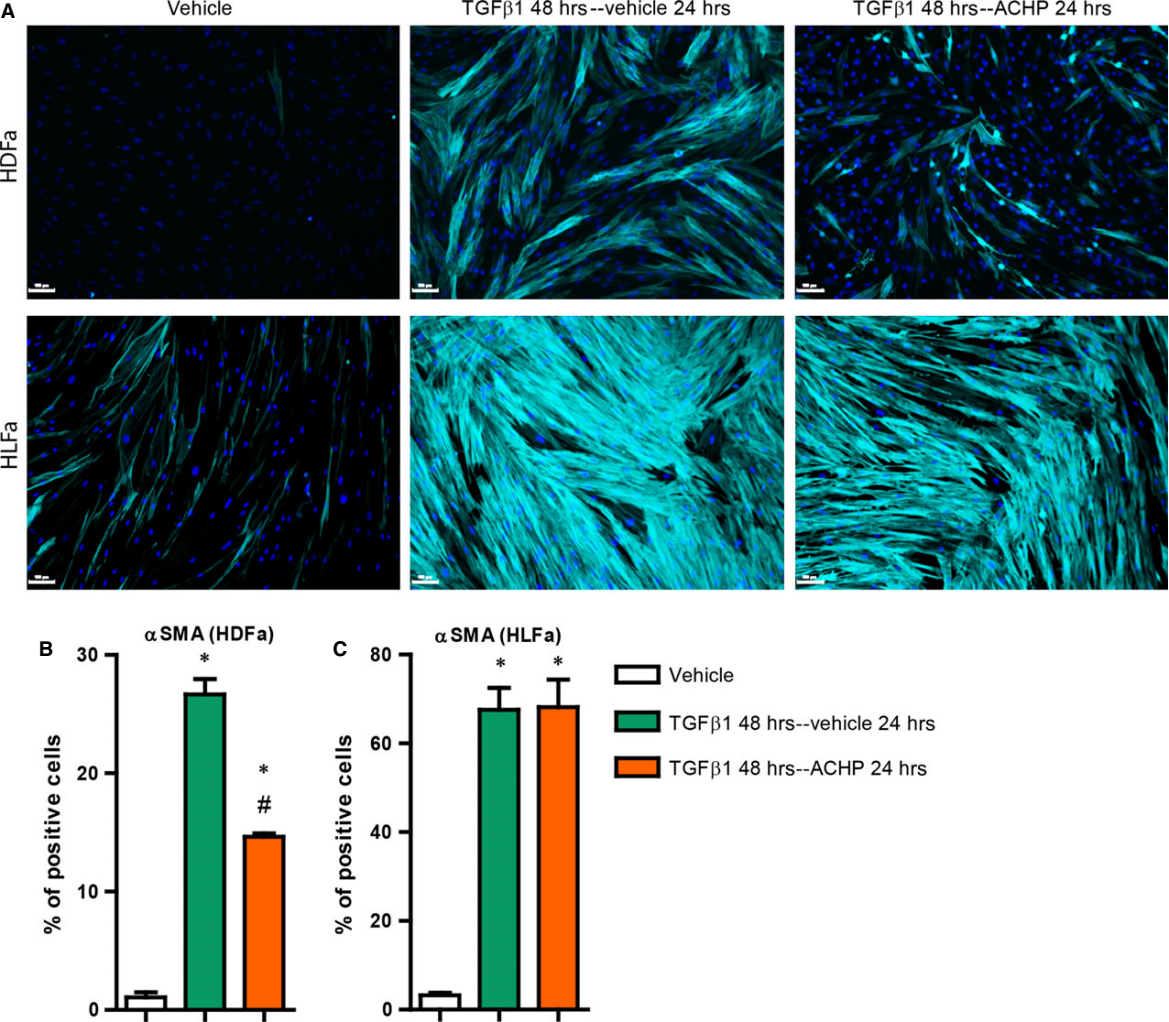
Post‐treatment effects of ACHP on fibroblasts treated with transforming growth factor beta1 (TGFβ1) in regard to alpha‐smooth muscle actin (αSMA) synthesis. Human dermal and lung fibroblasts (HDFa and HLFa) were cultured for 48 hrs in the presence of TGFβ1, followed by a post‐treatment with ACHP for 24 hrs. (A and B) representative stainings ad quantification of the % of cells positive for αSMA. The scale bars represent 100 μm. The sign * represents statistically significance towards untreated control, and the sign # represents statistically significance of fibroblasts post‐treated with ACHP compared to TGFβ1‐stimulated fibroblasts.

Myofibroblasts are the crucial cell types involved in ECM production and tissue contraction. These cells are enriched with cytoplasmic stress fibres containing αSMA and smooth muscle protein 22‐alpha (SM22α) 1, 2, 3, 4, 28. To examine whether ACHP can inhibit the TGFβ1‐induced differentiation of fibroblasts into myofibroblasts, HDFa and HLFa were incubated either with ACHP, TGFβ1 or both. Gene expression analysis of TGFβ1‐stimulated fibroblasts showed an up‐regulation (~7‐fold) in mRNA level of *ACTA2* (the gene that encodes for the protein αSMA) compared to unstimulated fibroblasts (Fig. 2A and B). Similarly, the immunofluorescence staining on αSMA protein showed an increase in the number of αSMA‐positive cells (in HDFa ~12% and in HLFa ~70%) after stimulation with TGFβ1. Interestingly, the combination of ACHP and TGFβ1 resulted in a decrease in αSMA synthesis to baseline levels, both on a gene and on a protein level. Treatment of ACHP alone also reduced the mRNA and protein level of αSMA in unstimulated HLFa, whereas ACHP had no effect on HDFa (since there are hardly αSMA‐positive cells detected in the pool of unstimulated HDFa) (Fig. 2A–E). These results reveal that ACHP is able to inhibit both the pre‐existing and the TGFβ1‐induced synthesis of αSMA stress fibres. In addition, to evaluate whether ACHP is able to reverse TGFβ1‐induced formation of αSMA, HDFa and HLFa were pre‐treated with TGFβ1 followed by ACHP incubation. TGFβ1‐treatment alone resulted in an increase in positively stained αSMA fibroblasts (in HDFa ~25% and in HLFa ~65%) and the post‐treatment with ACHP showed a ~50% reduction in αSMA‐positive cells in HDFa, whereas HLFa did not show such a decreasing effect on αSMA stress fibres (Fig. 3).

### Effect of TGFβ1 and ACHP on SM22α synthesis

**Figure 4:**
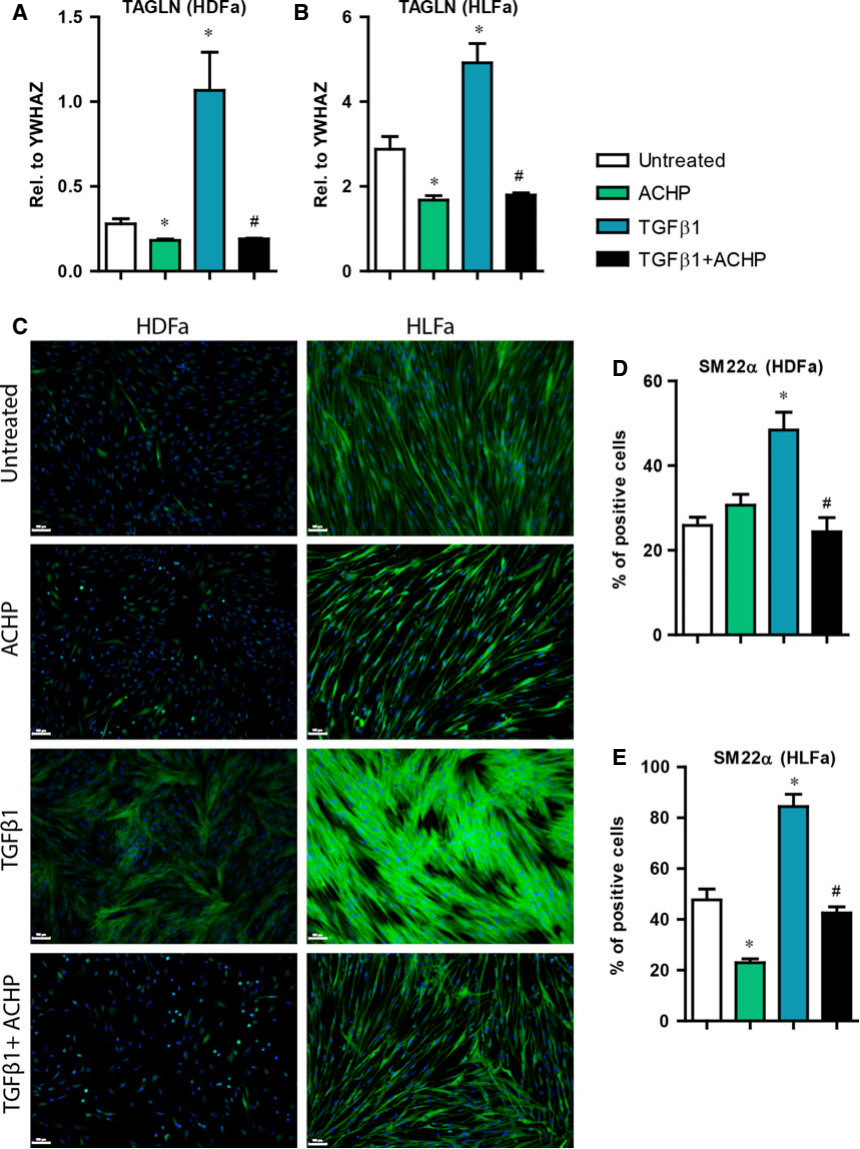
Effects of ACHP on transforming growth factor beta1 (TGFβ1)‐induced SM22α synthesis. (**A** and **B**) human dermal and lung fibroblasts (HDFa and HLFa) were cultured for 24 hrs in the presence of ACHP alone, TGFβ1 alone or TGFβ1 in combination with ACHP (co‐treatment). mRNA levels of *TAGLN* relative to the reference gene *YWHAZ*. (**C**–**E**) HDFa and HLFa were cultured for 48 hrs in the presence of ACHP alone, TGFβ1 alone or TGFβ1 in combination with ACHP (co‐treatment). Representative immunofluorescence stainings (left panel) and quantification of the % of cells (right panel) positive for SM22α. The scale bars represent 100 μm. The sign * represents statistically significance towards untreated control, and the sign # represents statistically significance of cells co‐stimulated with TGFβ1 and ACHP compared to TGFβ1‐treated cells.

Myofibroblasts are also characterized by increased levels of SM22α, another component of cytoplasmic stress fibres 28. Like αSMA, we investigated whether ACHP is able to prevent the synthesis of SM22α in a TGFβ1‐enriched environment. Compared to unstimulated fibroblasts, stimulation of HDFa and HLFa with TGFβ1 showed an up‐regulation of *TAGLN* mRNA (in HDFa ~4‐fold and in HLFa ~2‐fold), the gene that encodes for the protein SM22α. As was the case for *ACTA2*, this up‐regulation was blocked by ACHP to baseline levels both in HDFa and HLFa (Fig. 4A and B). The protein expression of SM22α showed a similar result as seen with mRNA expression. In comparison to untreated fibroblasts, SM22α stress fibres were up‐regulated (~2‐fold) with TGFβ1 for both fibroblast types; co‐treatment with ACHP suppressed the number of SM22α‐positive cells to baseline levels, both for HDFa and HLFa. Incubation of ACHP alone revealed a downregulation of SM22α synthesis both at the gene and protein level in HLFa, whereas in HDFa a reduction was seen only at mRNA level (Fig. 4C–E). These data show, together with the above data on αSMA, that ACHP is able to block the TGFβ1‐induced formation of myofibroblasts.

### Effect of TGFβ1 and ACHP on collagen type I synthesis

**Figure 5:**
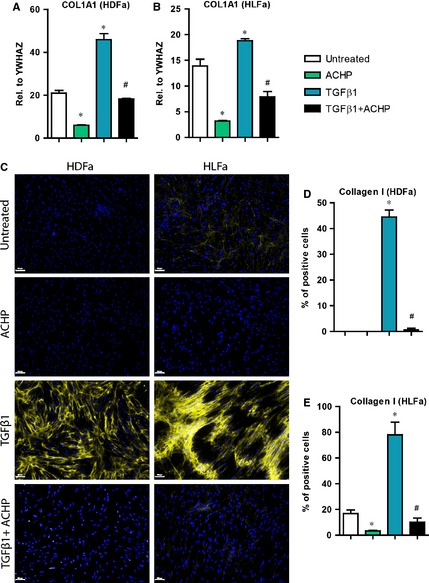
Effects of ACHP on transforming growth factor beta1 (TGFβ1)‐induced collagen type I synthesis. (**A** and **B**) human dermal and lung fibroblasts (HDFa and HLFa) were cultured for 24 hrs in the presence of ACHP alone, TGFβ1 alone or TGFβ1 in combination with ACHP (co‐treatment). mRNA levels of *COL1A1* relative to the reference gene *YWHAZ*. (**C**–**E**) HDFa and HLFa were cultured for 48 hrs in the presence of ACHP alone, TGFβ1 alone or TGFβ1 in combination with ACHP (co‐treatment). Representative immunofluorescence stainings (left panel) and quantification of the % of cells (right panel) positive for collagen I. The scale bars represent 100 μm. The sign * represents statistically significance towards untreated control, and the sign # represents statistically significance of cells co‐stimulated with TGFβ1 and ACHP compared to TGFβ1‐treated cells.

**Figure 6:**
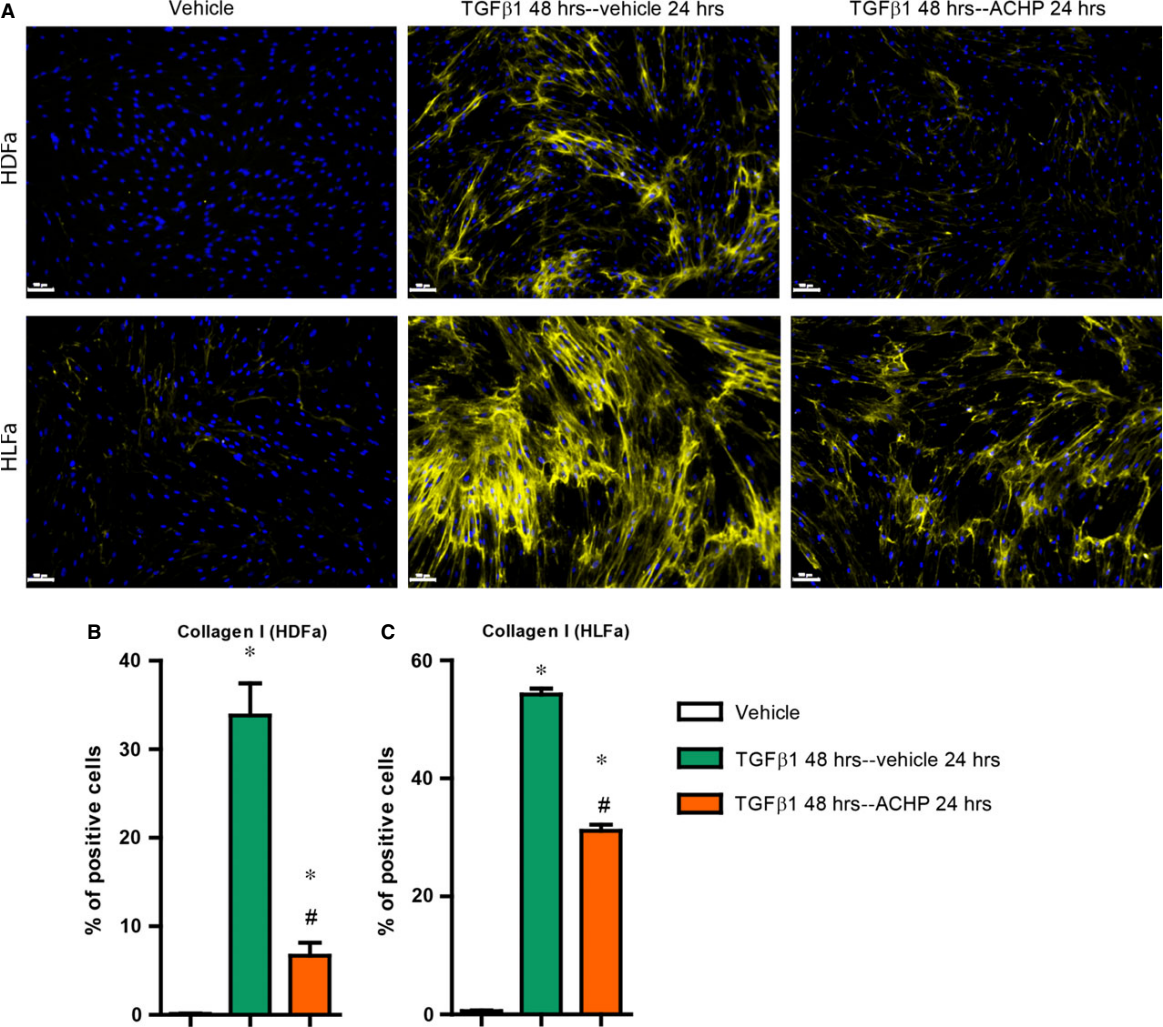
Post‐treatment effects of ACHP on fibroblasts treated with transforming growth factor beta1 (TGFβ1) with regard to collagen type I synthesis. Human dermal and lung fibroblasts (HDFa and HLFa) were cultured for 48 hrs in the presence of TGFβ1, followed by a post‐treatment with ACHP for 24 hrs. (A and B) representative stainings ad quantification of the % of cells positive for collagen type I. The scale bars represent 100 μm. The sign * represents statistically significance towards untreated control, and the sign # represents statistically significance of fibroblasts post‐treated with ACHP compared to TGFβ1‐stimulated fibroblasts.

Increased production and deposition of collagen type I by myofibroblasts is the main feature of fibrosis 29, 30. We investigated whether ACHP was able to block the synthesis of collagen type I. In TGFβ1‐stimulated fibroblasts an increase in mRNA level of *COL1A1* (the gene that encodes the α1 chain of collagen type I) is seen in either type of fibroblasts (~2.5‐fold in HDFa and ~1.5‐fold in HLFa) compared to untreated controls. Combination treatment of ACHP with TGFβ1 suppressed the expression of *COL1A1* to baseline levels both for HDFa and HLFa (Fig. 5A and B). A similar outcome was observed regarding collagen type I protein synthesis. Compared to untreated fibroblasts, a ~50% increase in the number of collagen‐producing cells was observed after TGFβ1‐stimulation, whereas co‐stimulation with ACHP blocked the synthesis of collagen type I to baseline for both fibroblast types (Fig. 5C–E). ACHP alone also inhibited mRNA expression of *COL1A1* in unstimulated HDFa and HLFa (Fig. 5A and B). Protein expression of collagen type I was completely abolished by ACHP in HLFa, but such an effect could not be observed in HDFa as there are hardly any cells positive for collagen type I expression in the unstimulated pool (Fig. 5C–E). Post‐treatment with ACHP after TGFβ1 treatment resulted in a major decrease (~30% in HDFa and ~25% in HLFa) of the number collagen‐expressing fibroblasts (Fig. 6). Taken together, these findings indicate that ACHP can at least slowdown the accumulation of collagen type I.

### Effect of TGFβ1 and ACHP on fibronectin synthesis

**Figure 7:**
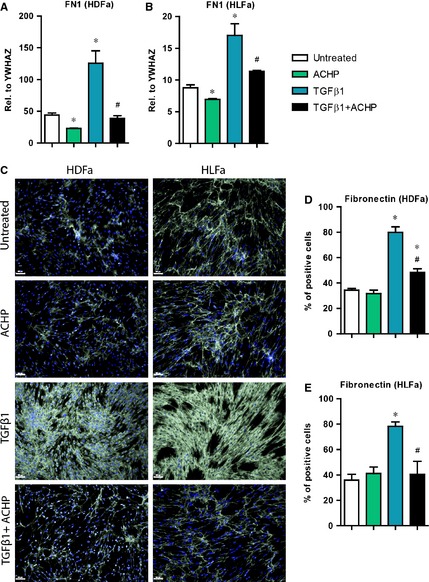
Effects of ACHP on transforming growth factor beta1 (TGFβ1)‐induced fibronectin synthesis. (**A** and **B**) human dermal and lung fibroblasts (HDFa and HLFa) were cultured for 24 hrs in the presence of ACHP alone, TGFβ1 alone or TGFβ1 in combination with ACHP (co‐treatment). mRNA levels of *FN1* relative to the reference gene *YWHAZ*. (**C**–**E**) HDFa and HLFa were cultured for 48 hrs in the presence of ACHP alone, TGFβ1 alone or TGFβ1 in combination with ACHP (co‐treatment). Representative immunofluorescence stainings (left panel) and quantification of the % of cells (right panel) positive for fibronectin. The scale bars represent 100 μm. The sign * represents statistically significance towards untreated control, and the sign # represents statistically significance of cells co‐stimulated with TGFβ1 and ACHP compared to TGFβ1‐treated cells.

The adhesion protein fibronectin (*FN1*) is an important ECM molecule required for the attachment and migration of fibroblasts and facilitates the construction of an organized matrix in tissue reparative processes. Aberrant expression of *FN1* under the influence of TGFβ1 contributes to fibrosis 2. Therefore, we have examined whether ACHP is able to prevent the TGFβ1‐induced production of fibronectin. Incubation of fibroblasts with TGFβ1 showed a ~3‐fold and ~2‐fold increase in the mRNA level of *FN1* in HDFa and HLFa, respectively. Co‐treatment with ACHP inhibited this up‐regulation, reaching baseline levels for both HDFa and HLFa (Fig. 7A and B). Immunofluorescent staining exhibited a 2‐fold increase in the number of fibronectin‐positive fibroblasts with TGFβ1 compared to untreated cells. TGFβ1 in combination with ACHP notably reduced the number of fibronectin‐positive cells, both for HDFa and HLFa (Fig. 7C–E). Treatment of fibroblasts with ACHP alone slightly inhibited the gene expression of *FN1*; this was not reflected in protein expression level for either type of fibroblasts (Fig. 7A–E). These results show that ACHP has an inhibitory effect on the synthesis of fibronectin.

### Effect of TGFβ1 and ACHP on the synthesis of members of the PLOD family

**Figure 8:**
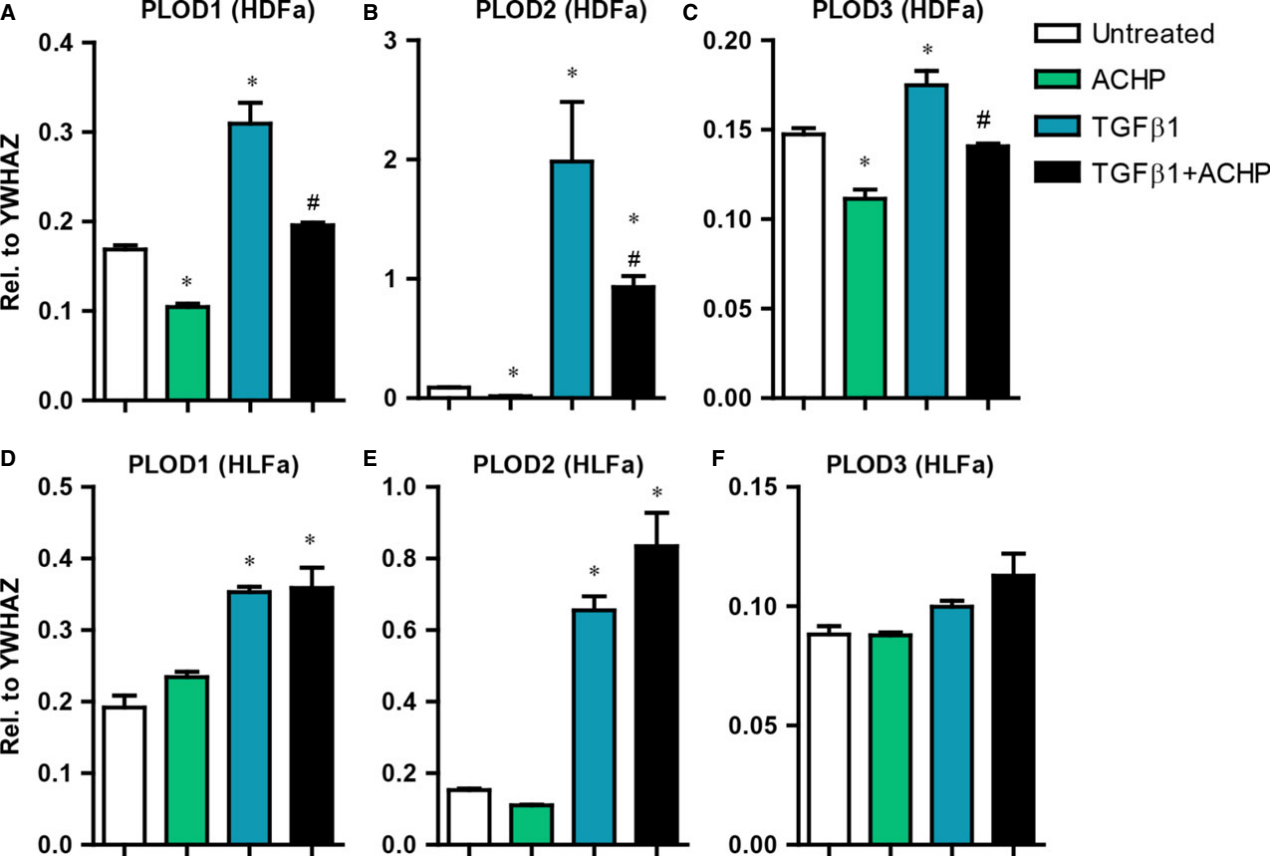
Effects of ACHP, transforming growth factor beta1 (TGFβ1) and TGFβ1+ CAPE on mRNA levels of *PLOD1*,*PLOD2* and *PLOD3*. (**A**–**F**) Human dermal and lung fibroblasts (HDFa and HLFa) were cultured for 24 hrs in the presence of ACHP alone, TGFβ1 alone or TGFβ1 in combination with ACHP (co‐treatment). mRNA levels of *PLOD1*,*PLOD2* and *PLOD3* relative to the reference gene *YWHAZ*. The sign * represents statistically significance towards untreated control, and the sign # represents statistically significance of cells co‐stimulated with TGFβ1 and ACHP compared to TGFβ1‐treated cells.

The processing and modification of collagen by lysyl hydroxylase (LH) enzymes plays a critical role in the stability of the collagen fibril. In fibrosis, a highly elevated level of the collagen‐modifying enzyme LH2 encoded by the gene *PLOD2* has been reported 31, 32, 33, 34, whereas up‐regulation of LH1 (encoded by *PLOD1*) and LH3 (encoded by *PLOD3*) is more modest. We wondered whether ACHP has an effect on the expression of the *PLOD* family members in our experimental setting. Compared to unstimulated cells, TGFβ1‐stimulation significantly increased mRNA levels of *PLOD1* and *PLOD2* in both fibroblast types; the fold‐increase for *PLOD2* was always higher than for *PLOD1*. Little or no increase in *PLOD3* expression was seen in HDFa and HLFa, respectively. The presence of ACHP during TGFβ1 incubation showed a downregulation of all three *PLOD*s in HDFa, whereas no down‐regulation was seen in HLFa. Similar observations were made when unstimulated fibroblasts were treated with ACHP alone (Fig. 8A–F).

### Effects of ACHP on proliferation of myo(fibroblasts)

**Figure 9:**
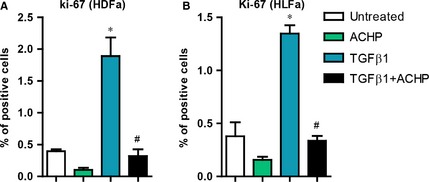
Effects of ACHP on the proliferation of myo(fibroblasts). (**A** and **B**) Human dermal and lung fibroblasts (HDFa and HLFa) were cultured for 48 hrs in the presence of ACHP alone, transforming growth factor beta1 (TGFβ1) alone or TGFβ1 in combination with ACHP (co‐treatment). Quantification of the % of cells positive for Ki‐67. The sign * represents statistically significance towards untreated control, and the sign # represents statistically significance of cells co‐stimulated with TGFβ1 and ACHP compared to TGFβ1‐treated cells.

Lastly, we investigated the effects of ACHP on the proliferation of myo(fibroblasts). Staining for the proliferation marker Ki‐67 showed that there was a significant increase in the proliferation of HDFa and HLFa upon TGFβ1‐stimulation compared to untreated fibroblasts. ACHP markedly reduced the number of TGFβ1‐induced Ki‐67 positive cells in both HDFa and HLFa. However, no difference was observed between the proliferation rates of untreated cells and cells treated with ACHP alone (Fig. 9A and B), indicating that the given concentration of ACHP is not toxic for the cells (as can also be concluded from the normal morphological appearance of the ACHP‐treated cells).

## Discussion

The strong pro‐fibrotic effects of TGFβ1 towards fibroblasts results in the formation of myofibroblasts, being the critical cell type that plays a central role in fibrosis. Myofibroblasts are responsible for the synthesis of large amounts of ECM 1, 2, 3, 4, 28. Here, we show that ACHP is able to completely abrogate the TGFβ1‐induced differentiation of both dermal and lung fibroblasts into myofibroblasts, as revealed by the inhibition of the formation of the stress fibre components αSMA and SM22α. The data on the post‐treatment by ACHP revealed a 50% decrease in TGFβ1‐induced αSMA‐expressing dermal fibroblasts, whereas no decrease was observed in lung cells. These findings indicate that ACHP is able to inhibit myofibroblast formation of HDFa and HLFa in a TGFβ1‐rich environment. In the case of HDFa, ACHP was even able to partially reverse myofibroblast into fibroblasts. The diminished nuclear translocation of NFκB, as induced by ACHP, has apparently major implications on the TGFβ pathway itself. Indeed, we showed also a lack of nuclear translocation of Smad2/3, a downstream target of TGFβ, thus providing further mechanistic evidence on how NFκB regulates myofibroblast differentiation 5, 9, 19.

Excessive collagen production is the hallmark of fibrosis 29, 30. We have found that ACHP blocks the synthesis of TGFβ1‐induced collagen type I to baseline values, both at gene and protein level, and independent of fibroblast type. A considerable reduction in synthesized collagen type I protein was also detected in the case of post‐treatment with ACHP in both HDFa and HLFa. This is of interest, as post‐treatment with ACHP reduced αSMA in HDFa only, not in HLFa. Thus, ACHP is not only able to inhibit or reverse myofibroblast formation; it is also able to inhibit collagen synthesis, even in cells that still show myofibroblast properties. The HLFa data show that a downregulation in collagen type I synthesis is not automatically accompanied by a reduction in αSMA expression. These data are consistent with the opposite observation, namely that myofibroblasts which are negative for αSMA are still capable of synthesizing large quantities of collagen 31.

The collagen in fibrotic tissue has a higher level of pyridinoline cross‐links compared to collagen in normal tissue 32, 33, 34. This is because of the higher level of lysyl hydroxylation in the telopeptides of collagen. There are three LHs that convert lysine into hydroxylysine: LH1, LH2 (encompassing the splice variants LH2a and LH2b) and LH3. The telopeptides are hydroxylated by LH2b, whereas LH1 and LH3 hydroxylate certain Lys residues in the triple helical part of the collagen molecule 35. The substrate specificity of LH2a is unknown, but most likely not the telopeptides. A higher expression of LH2 has been reported in fibrosis (specifically: LH2b) 32, 33, 34, 36, explaining the higher level of pyridinoline cross‐links. How the expression of *PLOD1*,* PLOD2* and *PLOD3* (encoding LH1, LH2 and LH3 respetively) is regulated is essentially unknown. We therefore investigated the effect of ACHP on expression levels of the three LHs. ACHP inhibited the expression of all three *PLOD*s in HDFa, whereas no such inhibition was seen in HLFa. It thus seems the regulatory pathways that result in the expression of the three LHs differs, at least in part, between HDFa and HLFa.

In conclusion, the main message of this study is that ACHP exhibit strong anti‐fibrotic properties. It is able to directly abolish the TGFβ1‐induced myofibroblast formation and the synthesis of ECM components collagen type I and fibronectin. It also has the ability to partly reverse existing myofibroblasts into fibroblasts and the subsequent deposition of collagen type I. By inhibiting IKK, the NF‐κB/IκB complex remains in the cytoplasm, preventing the translocation of NF‐κB to the nucleus. The data obtained with ACHP shows that the NF‐κB pathway play an important role in promoting fibrosis.

## Conflicts of interest

The authors declare that they have no conflict of interest.
